# Adhesion properties of toxigenic corynebacteria

**DOI:** 10.3934/microbiol.2018.1.85

**Published:** 2018-02-09

**Authors:** Lisa Ott

**Affiliations:** Friedrich-Alexander-Universität Erlangen-Nürnberg, Professur für Mikrobiologie, Staudtstr. 5, 91058 Erlangen, Germany

**Keywords:** *Corynebacterium diphtheriae*, *Corynebacterium pseudotuberculosis*, *Corynebacterium ulcerans*, bacterial adherence, epithelial cells, niche factors

## Abstract

*Corynebacterium diphtheriae*, *Corynebacterium pseudotuberculosis* and *Corynebacterium ulcerans* share one distinctive feature: they are all putative carriers of the diphtheria toxin (DT), encoded by a β-corynephage integrated into the genome. Due to its medical relevance, *C. diphtheriae* may be the most highly investigated species of the genus *Corynebacterium*. Nevertheless, systemic infections caused by *C. ulcerans* are increasingly being reported indicating that this specie*s* is an emerging pathogen today. *C. diphtheriae*, *C. pseudotuberculosis* and *C. ulcerans* are able to colonize different types of epithelial cells in a strain-specific manner, independent of the presence of the *tox* gene. However, the molecular mechanisms contributing to host colonization are barely understood. This review gives a comprehensive update of recent data concerning the adhesion properties of toxigenic corynebacteria, demonstrating that adhesion is a multi-factorial process.

## 1. Introduction

*Corynebacterium diphtheriae*, *Corynebacterium pseudotuberculosis* and *Corynebacterium ulcerans* are, among others, pathogenic species of the genus *Corynebacterium*. Corynebacteria belong to the family *Corynebacteriaceae* within the order *Actinomycetales* and the phylum *Actinobacteria* [1]. *Corynebacterium* species can be found naturally in soil and water and are able to colonize the skin and mucous membranes of animals and humans. All are Gram-positive, aerobic, irregular or clubbed-shaped bacteria with high G + C content DNA [2],[3]. The cell envelope of these bacteria comprises a plasma membrane covered by a peptidoglycan layer, which is covalently bound to arabinogalactan. On top of this, an additional outer layer of mycolic acids is found, which is functionally equivalent to the outer membrane of Gram-negative bacteria [4]–[7]. Corynebacteria are closely related to members of the genus *Mycobacterium*, *Nocardia* and *Rhodococcus*, which are referred to as the CMNR group [8]–[11]. These organisms are characterized by a complex cell wall structure, and high G + C content DNA. In contrast to *Mycobacterium tuberculosis*, corynomycolic acids may not be crucial in *Corynebacterium* host interaction [12]. Therefore, in corynebacterial pathogenicity, other determinants may have functions such as those of the trehalosyl-dimycolates of *M. tuberculosis*.

*C. diphtheriae* is the classical etiological agent of diphtheria, an inflammatory disease of the upper respiratory tract, but also cases of cutaneous diphtheria occur. Additionally, systemic infections such as endocarditis, osteomyelitis, pneumonia and others are increasingly being reported [13]–[15]. Due to vaccination programs diphtheria is well controlled in Western countries but not totally eradicated [16]–[18]. Even after more than 20 years since the last large-scale outbreak in 1990s in the former states of the Soviet Union, diphtheria remains a severe public health problem, not only in less developed countries ([13],[19] and references therein).

**Figure 1.**
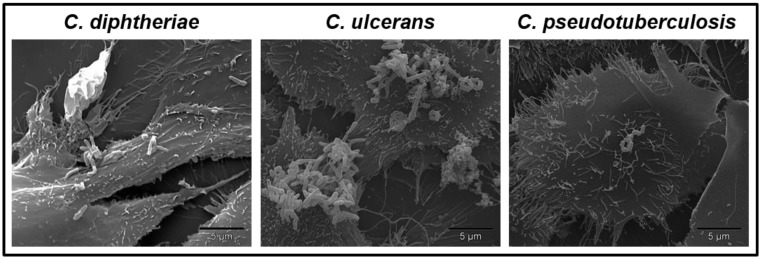
SEM of *C. diphtheriae* ISS4060, *C. ulcerans* BR-AD22 and *C. pseudotuberculosis* FRC41 adhered to Detroit562 cells. Infection of Detroit562 cells with three different corynebacteria species for 90 min under cell culture conditions. Subsequently, cultures were fixed, dehydrated, sputtered with gold and examined using an FEI Quanta 200 scanning electron microscope (L. Ott, unpublished data).

*C. pseudotuberculosis* and *C. ulcerans* are as is *C. diphtheriae* potential carriers of the diphtheria toxin. The strains can be infected by *tox* gene-carrying corynebacteriophages that integrate into the bacterial genome [20]–[23]. Since the ability to produce the diphtheria toxin is a result of β-corynephages infection, for each species toxigenic as well as non-toxigenic strains exist. Regardless of the presence of the *tox* gene, all three species are able to adhere to epithelial cells in a strain-specific manner (Figure 1, unpublished data).

*C. pseudotuberculosis* causes caseous lymphadenitis in sheep and goats resulting in skin lesions and abscessed tissues with the consequence of severe economic losses in production of meat, milk and wool [24]–[26]. Additionally, *C. pseudotuberculosis* has also been isolated from horses, cattle, camels, buffalo and humans and zoonotic transmission can be assumed [26]. To date, no detailed information is available as to how this organism is able to adhere to host cells or how it gains access to deeper tissues. The most prominent virulence factor of *C. pseudotuberculosis* may be phospholipase D (PLD), a sphingomyelinase D that catalyzes the hydrolysis of phosphatidylcholine and cleaves ester bonds in sphingomyelins that are essential components of eukaryotic membranes [27]–[29]. Although *C. pseudotuberculosis* can harbor the DT encoding gene, no cases of classical respiratory or cutaneous diphtheria in humans caused by this species have been reported up to now.

*C. ulcerans* was first isolated in 1927 from a patient with respiratory diphtheria-like illness [30], but this pathogenic bacterium has a wide range of hosts. *C. ulcerans* is commensal in wild and domestic animals and can cause mastitis in cattle [31]–[33]. Human infections with *C. ulcerans* are typically associated with the consumption of raw milk products or contact with farm animals, but also domestic animals such as cats and dogs are able to transmit *C. ulcerans* infections to humans [34]–[36]. Person-to-person transmission is rare, but cannot be excluded [37],[38]. As already mentioned above, *C. ulcerans* can carry the gene encoding DT, and in contrast to *C. pseudotuberculosis*, this pathogen can cause classical respiratory as well as cutaneous diphtheria in humans [39]. Interestingly, from 102 cases of infections by toxigenic corynebacteria that have been reported in the UK between 1986 and 2008, 59 of the patients were infected by *C. ulcerans*, 42 with *C. diphtheriae* and only one with *C. pseudotuberculosis* [40]. The occurrence of severe *C. diphtheriae* and *C. ulcerans* infections in Western countries shows the need to learn more about the infection process of these emerging pathogens. To date, a limited number of factors, which are involved in the adhesion process of toxigenic corynebacteria, are known. This review will give a comprehensive overview about the adhesion properties of three toxigenic *Corynebacterium* species, showing that adhesion is a multi-factorial process relying on specific and general mechanisms.

## 2. Adhesion, a multifactorial process

### 2.1. Effect of iron-limitation on bacterial adherence

It is known that iron restriction promotes lower growth rates of bacterial pathogens, but this may also stimulate these organisms to induce greater tissue damage, since the expression of many virulence factors is regulated by the iron supply in the environment. Iron limitation, for example, leads to slime production by *Staphylococcus aureus* [41] and *Staphylococcus epidermidis* [42], production of mucin-binding adhesins by *Pseudomonas aeruginosa* [43] in addition to hydrophobicity of and adherence to HEp-2 cells by *Vibrio parahaemolyticus* [44]. *C. diphtheriae* is also able to overcome low iron supply in the host cell, for example, by expressing genes encoding siderophores or other iron uptake systems [45],[46]. Studies by Moreira and co-workers (2003) investigated the influence of low iron availability on the expression of proteins and surface sugar residues of toxigenic *C. diphtheriae* strains, as well as their adherence to human B erythrocytes and HEp-2 cells. It was found that the production of cell wall surface carbohydrates is inversely correlated with the iron concentration of the culture media [47]. Further, the expression of sialidases, which work as glycosyl hydrolases catalyzing the cleavage of terminal sialic acid residues from a variety of glycoconjugates, also correlates with iron depression [47],[48]. In addition, by decorating their own cell surface with sialic acids from the host cell, the bacteria may be able to elude the host immune response that may otherwise rapidly clear an unsialyated strain [49]. Moreira's group identified a putative exo-sialidase NanH (DIP0543) in *C. diphtheriae* that shows *trans*-sialidase activity by using sialyl-α-2,3-lactose and sialyl-α2,6-lactose as donors [47]. NanH also presents a putative virulence determinant in *C. ulcerans* [50]. *C. ulcerans* produces high levels of sialidase when introduced into the skin of small rodents leading to rapidly purulent lesions [51].

In order to address the influence of iron limitation on adhesiveness, Moreira and co-workers cultivated two different *C. diphtheriae* strains, CDC-E8392 and 241, under iron-limited conditions (TSB-Fe). Strain 241 showed dramatically enhanced adhesion rates to HEp-2 cells in iron-limited medium, while strain CDC-E8392 showed no significant changes in adherence when cultivated in TSB-Fe medium, compared to standard TSB conditions. Moreover, a strongly reduced number of viable bacteria in TSB-Fe medium for strain CDC-E8293 were observed, which could be explained by medium sensitivity for this strain but needs further investigation [47]. However, no specific iron regulated genes that are involved in adherence of any toxigenic *Corynebacterium* species are characterized until now. In summary, the availability of iron in the culture medium seems to have a significant influence on the expression of a variety of pathogenic bacterial genes, and may therefore have an effect on the adhesion ability of microorganisms.

### 2.2. Lipoarabinomannan

The cell wall of *C. diphtheriae* is composed of branched long-chain mycolic acids and other lipoglycans such as lipomannans (LM) and lipoarabinomannans (LAM) [52]. Several studies discussed LAMs, such as the mannosylated LAM of *M. tuberculosis* (ManLAM), as virulence factors of pathogenic bacteria [53]. Besides the capacity to induce production of interleukin-12 and apoptosis in macrophages, ManLAM is able to induce granuloma formation of multinucleated giant cells [54],[55]. Moreira and co-workers were able to purify a LAM-like molecule from *C. diphtheriae* (CdiLAM) [56]. The detailed carbohydrate analysis of CdiLAM revealed an unusual substitution at position 4 of the α-1→6-mannan backbone by α-D-Araf. Variations in the substituting arabinan domains are already known, for example, for ReqLAM of *Rhodococcus equi*, RruLAM of *Rhodococcus ruber* and TotLAM of *Turicella otitidis* [57]–[59]. In the case of CdiLAM, Moreira and co-workers could not show hemagglutination of human B erythrocytes. Further, strain 241 showed reduced ability to adhere to HEp-2 cells when the bacteria were pre-incubated with rabbit polyclonal antibody anti-CdiLAM, and when HEp-2 cells were pre-incubated with purified CdiLAM, inhibited bacterial adherence was observed [56]. Based on these results, Moreira and co-workers postulated that the CdiLAM is acting as an adhesin only in the initial step of infection (30 min post infection), which leads then to the expression of additional bacterial adhesins or host cell receptors.

### 2.3. Function of pili in colonization of host cells

Proteinaceous filaments such as pili are known to play a role in bacterial adherence. Pili structures of Gram-positive bacteria are covalently linked to the peptidoglycan layer of the cell wall [60]. Genomic analysis of *C. diphtheriae* revealed the identification of three distinct pili clusters (*spaABC, spaDEF*, *spaGHI*) together with five sortase-encoding genes (*srtA–E*), which are essential for pilus assembly [61]. The major pilin proteins (SpaA, SpaD and SpaH, respectively) carry an LPxTG motif at the C-terminus, which is recognized by an additional housekeeping sortase, SrtF, for cell wall anchoring [62]–[64]. Mandlik and co-workers gave a detailed overview about the assembly and the function of pili in host colonization [65]. Briefly, by using a number of corynebacterial mutant strains in host cell interaction studies it was shown that only SpaA-type pili contribute to adherence of *C. diphtheriae* to the human pharyngeal carcinoma cell line Detroit 562 (D562). In contrast, SpaD and SpaH-type pili seem to be involved in adherence to the human laryngeal carcinoma cell line HEp-2 and the human lung carcinoma cell line A549 [66]. In the absence of the pilus shaft (SpaA), the minor pili SpaD and SpaH are linked directly to the surface due to their LPxTG motif, which is recognized by sortase SrtA. Similar results were obtained for other Gram-positive bacteria such as *Streptococcus agalactiae*, showing that adhesion to human lung and cervix epithelial cells is implemented by minor pilin proteins [67]. Interestingly, deletion of different pili subunits does not lead to the total loss of adhesion ability of the investigated strains, indicating that other factors may be involved in the adhesion process [66]. Furthermore, different *C. diphtheriae* isolates vary dramatically in their ability to adhere to human epithelial cells such as D562 cells [68]. In order to address these variations in adhesion rates, Ott and co-workers investigated the correlation between pilus expression and adhesion efficiency. Ultrastructural analysis of seven wild type strains revealed strain-specific differences in pili formation. Strain ISS4060 completely lacks pili structures, while strain ISS3319 possesses spike-like pili. Interestingly, both strains show comparable adhesion rates [68]. This indicates that pili formation and adhesion are not strictly coupled. Based on RNA hybridization and immunoblotting experiments, Ott and co-workers detected isolate-specific differences in the expression pattern of pili subunits. All strains seem to carry the genes for the *spaABC* cluster, but in contrast to strain NCTC13129, which expresses three pili clusters (*spaABC*, *spaDEF* and *spaGHI*), none of the investigated strains harbored the *spaDEF* cluster. Only two strains, ISS4746 and ISS4749, encode the genes for *spaABC* and *spaGHI*, thereby expressing a high level of *spaA* and *spaH*, which could be crucial for pili length [68]. Recent pan-genomic studies of 20 *C. diphtheriae* sequences by [69] confirmed the data of Ott et al. (2010) [68] and Trost et al. (2012) [70], showing that the numbers and organization of *spa* operons varies between strains [68]–[70]. Comparative analysis of new gene sequences of four strains (ISS3319, ISS4060, ISS4746, ISS4749) previously investigated by Ott and co-workers (2010) [68] revealed that the SpaD and SpaH gene clusters are present in all four strains, whilst an additional cluster SpaA is present in ISS4746 and ISS4749 [69]. This may correlate with the observed differences in number and length of the pili [68]. In strain ISS3319 only the SpaD cluster seems to be fully functional, which also may explain the relatively low number of pili in this strain, compared to ISS4749 [68],[69]. Based on observations by Ott and co-workers, later studies from Broadway and co-workers characterized 42 clinical isolates with regard to toxigenicity and pili expression by PCR and immunoblotting with antibodies directed against different pili subunits [71]. The strains were separated into seven groups, depending on the presence of the diphtheria toxin as well as the respective pilus gene. Broadway and co-workers' observations were similar to those of Ott and co-workers (2010) for different isolates. First, the presence of pilus genes varies considerably between different wild types; second, it is independent of the presence of the *tox* gene; and third, the SpaA-type is the pilus most represented in the investigated strains [68],[71]. In addition, Broadway and co-workers could confirm data reported previously by Mandlik and co-workers (2007) [66], which showed that isolates of subgroups that express SpaA-type pili exhibit significant adhesion to D562 cells [71]. Similar observations concerning the variations between pilus gene clusters were made for *C. pseudotuberculosis* in pan-genomic studies [72],[73]. Genetic analysis of two *C. ulcerans* isolates (BR-AD22 and 809) led to the identification of two gene clusters encoding adhesive pili structures as well as the genes coding for sortases SrtB and SrtC and the housekeeping sortase SrtF [50]. Compared to *C. diphtheriae* NCTC13129, one cluster is genetically identical to the *spaDEF* cluster, while another one lacks the genes for the major pilin subunit *spaA* of the *spaABC* cluster. To date, no detailed information is available about the expression and the role of pili subunits in the adhesion process of *C. ulcerans*. In summary, *C. diphtheriae*, *C. pseudotuberculosis* and *C. ulcerans* isolates exhibit a wide genetic diversity concerning the presence or/and expression of various pili gene clusters [50],[70],[73]. The reason for this still remains unclear. The fact that different pili types attach to different cell types in a specific manner indicates that this may be connected to different receptors on the host's surface or different animal reservoirs. However, pili seem to be specific adhesion factors, but they are not encoded only by pathogenic bacteria. Pilus proteins are also found in non-pathogenic *Corynebacterium* species such as *C. casei*, *C. efficiens*, *C. glutamicum* and others, and consequently seem not to be virulence factors strictly speaking.

### 2.4. The role of NlpC/P60 family proteins in adhesion

NlpC/P60 proteins belong to a large superfamily of proteins that are found in bacteria, RNA viruses, bacteriophages and eukaryotes. These proteins show a wide range of functions working as invasion-associated proteins, putative lipoproteins, cell wall hydrolases or putative endopeptidases [74]. Several members of this superfamily are conserved among corynebacteria. In this context, Hansmeier and co-workers initially predicted the function of DIP1621 as an invasion-associated protein, homologous to Cg2401 of *Corynebacterium glutamicum* [75]. By using Tn5 transposon mutagenesis of *C. diphtheriae* strain 225 and subsequent adhesion experiments with human epithelial cells (HEp-2), Kolodkina and co-workers identified one mutant that showed a strongly reduced adhesion efficiency compared to the wild type strain. Sequence analysis of the interrupted part of this mutant revealed the gene encoding DIP1621, indicating that this gene obviously might play a role in adhesion to epithelial cells [76]. Ott and co-workers made similar observations for another member of the NlpC/P60 family, DIP1281 [77]. The corresponding mutant strain showed reduced adherence to and invasion into the host cell. Unfortunately, the investigators were not able to generate a complementation strain, which might be due to the structural alterations on the surface of the mutant. The mutant exhibited an increased cell size and the formation of chains of cells. Furthermore, immunofluorescence microscopy with an antiserum directed against the surface proteome of *C. diphtheriae* showed an uneven, speckled staining of the mutant compared to the wild type. Due to these structural changes of the mutant strain, the reduced adhesion rate may be considered as a secondary effect [77]. In a pool of protein precursors from *C. ulcerans*, homologs of DIP1621 and DIP1281were found that represent putative molecules involved in adhesion of this microorganism, but thus far, no studies on further characterization exist [50].

### 2.5. Binding to extracellular matrix proteins

Proteins of the extracellular space of eukaryotic cells, such as collagen, fibronectin, laminin and elastin, constitute characteristic targets of microbial surface components recognizing adhesive matrix molecules (MSCRAMMs) of pathogenic bacteria [78]. It is assumed that the presence of collagen or hyaluronan in wounds may have significant influence on the attachment and biofilm formation of notorious biofilm producers, such as methicillin-resistant *P. aeruginosa* or *S. aureus* (MRSA) [79]. Also for *C. diphtheriae*, binding to collagen as well as to fibronectin was observed [80]. Peixoto and co-workers recently characterized the protein DIP2093 with regard to its role in the virulence of *C. diphtheriae*. Bioinformatics analysis of this protein revealed structural similarity to SdrD protein from *S. aureus*, which plays an important role in the adherence of *S. aureus* to the extracellular matrix (ECM) and in biofilm formation. Furthermore, the DIP2093 sequence seems to be highly conserved among many strains of *C. diphtheriae*, *C. pseudotuberculosis* and *C. ulcerans* [81]. Peixoto and co-workers successfully generated a DIP2093 mutant strain, which was investigated in comparison to its parental strain NCTC13129 with respect to its influence on collagen-binding as well as its interaction with human epithelial cells. Peixoto and co-workers could show that DIP2093 is involved in binding to Type I, but not to Type IV, collagen [81]. The data also revealed that binding to Type I collagen was not totally reduced in the mutant strain, indicating that DIP2093 is not alone in acting as a MSCRAMM in *C. diphtheriae*. In order to address the role of DIP2093 in the adhesion process to epithelial cells, infection assays with HeLa cells were carried out. The mutant strain showed significantly reduced adhesion rates to HeLa cells compared to the wild type. Additionally, partial complementation of the mutation was achieved. To confirm these results, *in vivo* studies using mice as a mammalian model system were performed, showing that the mutant strain causes less severe symptoms in the carpus and no sign of inflammation in the tarsus. In contrast, the wild type strain NCTC13129 caused clear symptoms of arthritis and osteomyelitis [81]. This data demonstrated that the DIP2093 protein is not only involved in collagen-binding but also in host colonization.

Fibrinogen is one of the major proteins of human plasma. The enzymatic cross-linking of fibrinogen to fibrin monomers leads to the polymerization of fibrin that covers a wound site. Fibrinogen synthesis is upregulated during inflammation or systemic infections; therefore, it is conceivable that human pathogenic organisms are able to interact with those proteins [82]. Additionally, another protein that is found in soluble form in many blood fluids and in an insoluble form on cells surfaces, membranes and extracellular matrices is fibronectin. Fibronectin is able to bind fibrinogen, fibrin, collagen, human and bacterial cells and might serve as a receptor in bacterial adherence [83]–[86]. Simpson-Louredo and co-workers performed investigations on *C. ulcerans* concerning its ability to bind human plasma fibrinogen, fibronectin and Type I collagen [87]. The strains used for this study were isolated from a human fatal pulmonary infection and asymptomatic dogs. The investigated strains were able to bind to fibrinogen, fibronectin and Type I collagen in a strain-specific manner. Thereby, the human isolate *C. ulcerans* 809 showed the lowest ability to bind the ECM and plasma proteins, while strain BR-AD22, isolated from a dog, exhibited the highest levels of binding to ECM/plasma proteins [87]. One reason might be differences in qualitative and quantitative expression of bacterial adhesins that bind fibrinogen, fibronectin and Type I collagen, leading to variations in the virulence potential of the bacterium [80].

### 2.6. Multi-functional protein DIP0733

In order to address the molecular mechanism of adherence in more detail, a protein of *C. diphtheriae* known as non-fimbrial protein 67-72p became a focal point of research for several groups. Colombo and co-workers (2001) first described this protein as 67-72p, since two polypeptide bands of 67 and 72 kDa were detected by Western blot analysis. Both polypeptides are able to bind human erythrocytes receptors [88]. By using anti-67-72p IgG antibodies, the binding not only to human erythrocytes but also to HEp-2 cells (human epidermoid laryngeal carcinoma cells) was effectively blocked [88],[89]. In 2012, Sabbadini and co-workers performed comparative MALDI-TOF MS analysis of 67-72p from strain CDCE-E8392 with an *in silico* proteome of this strain. This led to the identification of 67-72p as DIP0733 protein, which was initially a hypothetical protein. DIP0733 comprises seven aminoterminal transmembrane helices and a long carboxyterminal segment [90]. A corresponding mutant strain was characterized in host-pathogen interaction studies [91]. The mutant showed a significantly reduced ability to adhere to HeLa cells compared to the parental strain CDC-E8392. Additionally, the complementation strain reached a 7-fold higher adhesion rate to epithelial cells than the corresponding empty vector control strain. In a nematode infection model system, the DIP0733 mutant lost the ability to colonize the gut of *C. elegans* almost completely, while the wild type strain showed persistent colonization all over the gut. Finally, Antunes and co-workers (2015) confirmed data from Sabbadini and colleagues that *C. diphtheriae* can interact with collagen and fibrinogen [80],[91]. They provided evidence that DIP0733 protein is involved in these processes but is not exclusively responsible for it. Due to its multi-functional properties, DIP0733 plays an important role in the virulence of *C. diphtheriae* and needs to be further investigated. In contrast, the function of the DIP0733 homolog CULC22_00609 of *C. ulcerans* BR-AD22 is barely understood. Hacker and co-workers were able to generate a CULC22_00609 mutant strain, which showed no significant difference in its interaction with epithelial cells compared to the wild type [92].

### 2.7. Colonization of epithelial cells

Many data exist concerning the ability of *C. diphtheriae* wild type strains to interact with different epithelial cells, such as HeLa, HEp-2, D562 and A549, but only a few proteins involved in the adhesion process are known thus far (Table 1).

**Table 1.** Experimentally verified factors involved in the adhesion process of *C. diphtheriae*.

Name	Function	Reference
SpaA type pili	adherence to HEp-2, A549 and D562 cells	[66],[68]
NanH (DIP0543)	sialidase activity	[47],[93]
CDiLAM	adherence to HEp-2 cells	[56]
DIP1621	adherence to HEp-2 cells	[76]
67-72p (DIP0733)	hemagglutination	[88],[89],[91]
	adherence to HEp-2 cells and HeLa cells	
	collagen and fibrinogen-binding	
	colonization of *C. elegans*	
DIP1546	colonization of *C. elegans*	[94]
	adherence to D562 cells	
DIP2093	binding to type I collagen	[81]
	adherence to HeLa cells	

**Figure 2.**
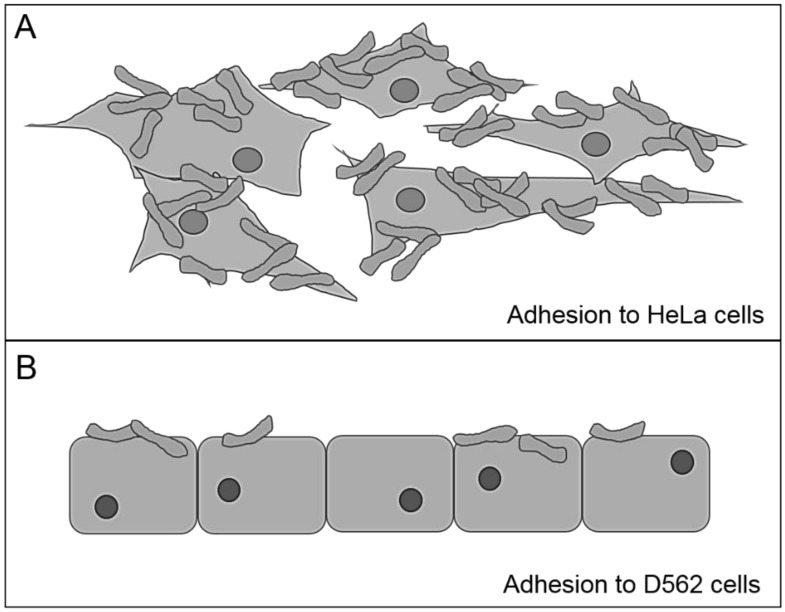
Adhesion pattern of *C. diphtheriae* to HeLa (A) and Detroit562 (D562) cells (B). *C. diphtheriae* shows not only strain-specific but also cell line specific adhesion rates [95]. The adhesion rate to Hela cells is up to 10-fold higher and the pattern is more localized compared to D562 cells. After 90 min of infection up to 70% of bacteria used for infection adhere to HeLa cells, while an adhesion rate of <8% to D562 cells is observable.

In a comparative analysis of the adhesion ability of different *C. diphtheriae* isolates to epithelial cells, the strains show strain-specific differences. Remarkably, the adhesion rates are not only strain specific but also cell line specific (Table 2 and Figure 2).

Thus, most strains show an up to 10-fold higher adhesion rate to HeLa cells than to D562 cells [95]. Since the molecules on the host's surface that interact with *C. diphtheriae* are largely unknown, further investigations have to carried out to identify not only potential adhesion factors of *C. diphtheriae* but also the receptor on the host's surface.

To the best of our knowledge, Hacker and co-workers published the first scanning electron microscopy as well as fluorescence microscopy images of *C. ulcerans* 809 and BR-AD22 infecting D562 and HeLa cells [92]. Independent of the cell line, the two strains showed a clustered adhesion pattern, which was not a consequence of bacterial aggregation in culture medium but may rather indicate accumulation of receptors in this area. Hirata and co-workers had already observed a similar type of adhesion pattern for toxigenic *C. diphtheriae* strains to HEp-2 cells [89]. Furthermore, Hacker and co-workers presented the first quantitative data that is available to date about the adhesion efficiency of *C. ulcerans* strains. Strain 809 showed comparable results independent of the cell line, while strain BR-AD22 exhibited a fivefold higher rate to HeLa cells compared to D562 cells. The data also revealed that the investigated strains are able to multiply during *in vitro* infection. In conclusion, the adhesion of *C. ulcerans* 809 is almost identical to both cell lines, whilst strain BR-AD22 shows strain-specific and cell line-specific adhesion rates [92]. Compared to *C. diphtheriae*, *C. ulcerans* adhesion to epithelial cells is up to fivefold higher, which probably hints at the high virulence potential of *C. ulcerans*. However, the basic mechanism and specific virulence determinants that are involved in the adhesion process of *C. ulcerans* are unknown thus far.

**Table 2.** Strain-specific and cell line-specific adhesion rates of different *C. diphtheriae* wild type strains. Different cell lines were infected with *C. diphtheriae* wild type strains, washed, detached, lysed and CFU were determined. The ratio of bacteria used for infection (number of colonies on inoculum plates) and bacteria in the lysate (number of colonies on the lysate plates) multiplied by 100 gave the adhesion rate in percent. Values over 100% are the result of proliferating bacteria during infection.

Strain	Cell line	Time post infection [min]	Adhesion rate [%] (rounded values)	Reference
ATCC27010	HeLa	90	6	[96]
ATCC27012	HeLa	90	4	[96]
BR-INCA5015	HeLa	90	10	[96]
CDC-E8392	HEp-2	120	30	[89]
	HeLa	90	25	[91]
NCTC13129	A549	60	20	[66]
	D562	60	9	
	HEp-2	60	25	
	HeLa	90	9	[81]
241	HEp-2	120	30	[47]
ISS3319	D562	90	4	[95]
	HeLa	90	60	
ISS4060	D562	90	4	[95]
	HeLa	90	70	
ISS4746	D562	90	7	[95]
	HeLa	90	40	
ISS4749	D562	90	7	[95]
	HeLa	90	55	
DSM43988	D562	90	1	[95]
	HeLa	90	70	
DSM43989	D562	90	0.5	[95]
	HeLa	90	1	
DSM44123	D562	90	2	[95]
	HeLa	90	40	
INCA-402	D562	90	16	L. Ott, unpublished
	HeLa	90	160	

To date, not much data exists concerning adherence of *C. pseudotuberculosis* to host cells. Valdivia and co-workers first published results regarding the ability of three different *C. pseudotuberculosis* isolates from goats and sheep and one reference strain from the Spanish Type Culture Collection, to adhere to ovine embryonic kidney cells (FLK-BLV-044 cells) [94]. The strains showed no significant strain-specific difference in adhesion rates. In comparison with experiments carried out by Ott and co-workers with different *C. diphtheriae* isolates and D562 or HeLa cells, *C. pseudotuberculosis* showed significantly lower adhesion rates of approximately 1.5–2.0% [95],[97]. One reason may be, that FLK-BLV-044 cells are not the main target of *C. pseudotuberculosis*, and the usage of different cell lines could lead to deviating results, which could provide more information about the infection process of *C. pseudotuberculosis*.

### 2.8. Caenorhabditis elegans as an in vivo model system

The standard *in vivo* model systems that were successfully established for pathogenic corynebacteria, such as guinea-pigs and mice [15],[39],[98],[99], offer several disadvantages, for example, high costs, cumbersome handling and ethical issues. Therefore, the aim of Ott and co-workers was to identify a non-mammalian model system that allows high throughput screening and testing of mutant strains [94]. The small nematode *C. elegans* turned out to be an attractive organism substituting for small rodents for the study of pathogenic corynebacteria. In microscopic analysis, all of the investigated wild type strains of *C. diphtheriae*, *C. pseudotuberculosis* and *C. ulcerans* showed colonization of the gut of the worms, but the localization differed between the strains. Thus, strain *C. diphtheriae* ISS3319 was detected in the foregut (buccal cavity and pharynx) and the midgut, and strain *C. diphtheriae* DSM43988 was found in the midgut, *C. diphtheriae* DSM43989 and *C. ulcerans* 809 were localized in the hindgut of the worm. Strains *C. ulcerans* BR-AD22 and *C. pseudotuberculosis* FRC41 spread throughout the body [94],[100]. Additionally, nematode killing assays revealed strain-specific differences in the virulence of the strains. As proof of principle, Ott and co-workers tested two mutant strains, one transposon mutant inserted in gene *dip1546* of *C. diphtheriae* ISS3319, and the PLD mutant of *C. ulcerans* BR-AD22. The *dip1546* mutant was drastically impaired to colonize the gut of *C. elegans* when compared to its parental strain. This data was supported by additional adhesion assays to D562 cells, which resulted in an almost complete loss of the adhesion rate of the mutant strain. In contrast, the PLD mutant of *C. ulcerans* showed no changes in gut colonization of *C. elegans*, which was in accordance with data observed in adhesion assays of D562 cells [94].

As a second study, Antunes and co-workers tested the DIP0733 mutant strain in comparison to its parental strain CDC-E8392 in the *C. elegans* model [91]. Indeed, the mutant strain was only scarcely detectable, while the wild type strain was localized throughout the gut. The overexpression of DIP0733 in the mutant partially complemented this defect. This data was confirmed by the nematode killing assay, in which the wild type and overexpression strain exhibited a severe detrimental effect on the worms, while the mutant had almost no impact on their viability [91]. These results highlight once again the high virulence potential of the DIP0733 protein.

## 3. Conclusions

**Figure 3.**
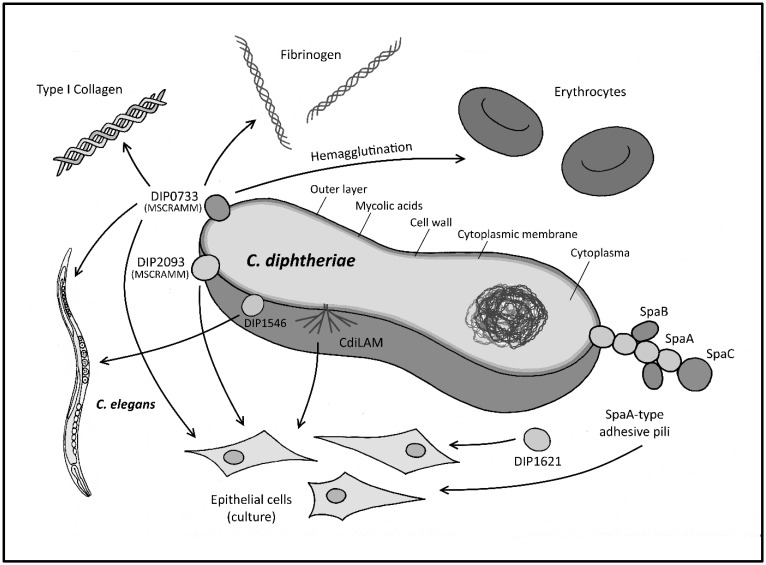
Adhesion of *C. diphtheriae*: a multi-factorial process. *C. diphtheriae* is able to bind different epithelial cell types in a strain-specific manner. A number of proteins involved in this process have been identified thus far, emphasizing the complexity of this event. Nevertheless, the deletion or disruption of one of these proteins, does not lead to a complete loss of the adhesion ability, indicating that a combination of proteins may be crucial for this event. *C. diphtheriae* carries genes for proteins that are termed as MSCRAMMS, due to their ability to mediate attachment to fibrinogen or collagen. Furthermore, *C. diphtheriae* is also able to bind human erythrocytes. Due to this capability, *C. diphtheriae* may be able to spread throughout the whole body via the blood stream.

Adhesion to the host cell was mainly considered to be the initial step in host-pathogen interaction. However, in the case of different *C. diphtheriae* isolates, it was shown that strains that are able to adhere to the host cell do not consequently invade the cell, and *vice versa* [95]. This indicates that adhesion and invasion are not coupled processes, and adhesion may not be obligatory for a pathogenic organism to display full virulence. As mentioned earlier, the pathogenicity of *C. diphtheriae*, *C. pseudotuberculosis* and *C. ulcerans* is not very well characterized at the molecular level. There are several factors known thus far that may contribute to adhesion to human tissues, but the lack of most of these proteins does not lead to the total loss of the adhesion ability. Figure 3 summarizes the known determinants that contribute to *C. diphtheriae* adherence.

These proteins might have a function in pathogenicity but work in combination with other proteins or toxins under certain environmental conditions. This makes sense considering that *Corynebacterium* species can be isolated from a wide range of ecological niches, such as synthetic surfaces, food, water, soil, animals and humans as well as others [1]. Therefore, adherence of pathogenic corynebacteria may not be a specific mechanism, but rather a more general event in host-pathogen interaction. In this regard, adhesion can be considered a multi-factorial process and proteins involved in this process can be designated as niche factors rather than virulence determinants [101],[102].
